# The CDK4/6 inhibitor LY2835219 has potent activity in combination with mTOR inhibitor in head and neck squamous cell carcinoma

**DOI:** 10.18632/oncotarget.7543

**Published:** 2016-02-21

**Authors:** Bo Mi Ku, Seong Yoon Yi, Jiae Koh, Yeon-Hee Bae, Jong-Mu Sun, Se-hoon Lee, Jin Seok Ahn, Keunchil Park, Myung-Ju Ahn

**Affiliations:** ^1^ Samsung Biomedical Research Institute, Seoul, Korea; ^2^ Division of Hematology-Oncology, Department of Medicine, Samsung Medical Center, Sungkyunkwan University School of Medicine, Seoul, Korea; ^3^ Division of Hematology-Oncology, Department of Internal Medicine, Inje University Ilsan Paik Hospital, Gyeonggi-do, Korea

**Keywords:** head and neck cancer, CDK4/6 inhibitor, mTOR, cell cycle, targeted therapy

## Abstract

Deletion of *CDKN2A* (p16) or amplification of *CCND1* (cyclin D1) occurs commonly in head and neck squamous cell carcinoma (HNSCC) and induces sustained cyclin-dependent kinase (CDK) 4/6 activation. Here, we report the antiproliferative activity of LY2835219, a selective CDK4/6 inhibitor through inhibition of CDK4/6-dependent Ser780 phosphorylation in retinoblastoma (RB) and induction of cell cycle arrest in HNSCC cells. In addition, we demonstrated the antitumor effects of HNSCC xenografts to LY2835219 *in vivo*. Given the limited effect in HNSCC as a single-agent treatment with LY2835219, a combinational strategy is required to enhance antitumor activity. At the molecular level, we found that LY2835219 inhibited activation of AKT and ERK, but not mTOR. The combination of LY2835219 with mTOR inhibitor was found to be more effective than either drug alone *in vitro* and *in vivo*. Taken together, our findings suggest that a combinational treatment with LY2835219 and mTOR inhibitor is a promising therapeutic approach for HNSCC.

## INTRODUCTION

Head and neck squamous cell carcinoma (HNSCC) refers to a large heterogeneous group of cancers of the lip, oral cavity, oropharynx, hypopharynx, and larynx [1, 2]. Locoregional recurrence and metastasis to lymph nodes occur in 40–60% of HNSCC and are associated with poor prognosis and low survival [3]. Despite recent advances in basic and clinical research, standard treatments for HNSCC including surgery, radiation or chemotherapy have not significantly improved survival rates [4]. Although cetuximab is the only approved targeted-therapy for HNSCC, the clinical use of cetuximab is usually limited by intrinsic or acquired resistance [5, 6]. Thus, there remain unmet medical needs for HNSCC and alternative treatment strategies are urgently needed.

Sustained proliferation is a hallmark of human malignancies. The retinoblastoma (RB) tumor suppressor plays a critical role in regulating cellular proliferation. The RB tumor suppressor pathway is frequently altered through deletion of *CDKN2A* (p16; ∼35%) or amplification of *CCND1* (cyclin D1; ∼94%) in HNSCC [2, 4, 7, 8]. Cyclin D-cyclin-dependent kinase 4/6 (CDK4/6) phosphorylates and inactivates RB, leading to release and activation of E2F transcription factors. E2Fs, in turn, initiate a transcription required for G1-S progression. Overexpression of cyclin D1 and resulting CDK4/6 hyperactivation are considered to be major oncogenic drivers [9]. p16, a potent inhibitor of CDK4/6, inhibits cyclin D1 interaction with CDK4/6 and prevents phosphorylation of the RB [10]. This sustains RB-mediated sequestration of E2F, thereby preventing cell cycle progression from the G1 to S phase. *CDKN2A* (p16) disruption has been reported as a frequent event in HNSCC and this alteration consequently promotes CDK4/6-mediated phosphorylation of RB [2, 11]. Thus, CDK4/6 inhibition can impede cell growth and targeting CDK4/6 with novel small-molecule inhibitor is one potential approach to treatment of HNSCC.

LY2835219 is an orally bioavailable drug that selectively inhibits CDK4/6 in the nanomolar range [12] and shows anti-proliferative activity in a number of tumor models *in vitro* and *in vivo* [13]. Antitumor activity of LY2835219 was also observed in colon cancer, lung cancer, glioblastoma, acute myeloid leukemia, and mantle cell lymphoma [13]. *In vivo*, LY2835219 inhibits CDK4/6 at clinically achievable plasma concentrations (∼400 nM), resulting in a G1 arrest that can be sustained with continuous oral dosing. Phase I study in advanced solid tumorsdemonstrated clinical efficacy at plasma concentrations of ∼562 nM [13]. Currently, phase II or III clinical studies of LY2835219 are ongoing in several malignancies including non-small cell lung cancer. However, the effect of LY2835219 on HNSCC has not been tested so far.

In this study, we explored the therapeutic effects of CDK4/6 inhibition, using the novel small-molecule CDK4/6 inhibitor, LY2835219 in both cell lines and tumor xenograft models of HNSCC. Our results show that CDK4/6 inhibition can have a potent antiproliferative effect on HNSCC, and specific combination with mTOR inhibitor significantly increases the therapeutic potency of LY2835219 in HNSCC.

## RESULTS

### LY2835219 decreases cell growth and induces cell cycle arrest in HNSCC cell lines

**Figure 1:**
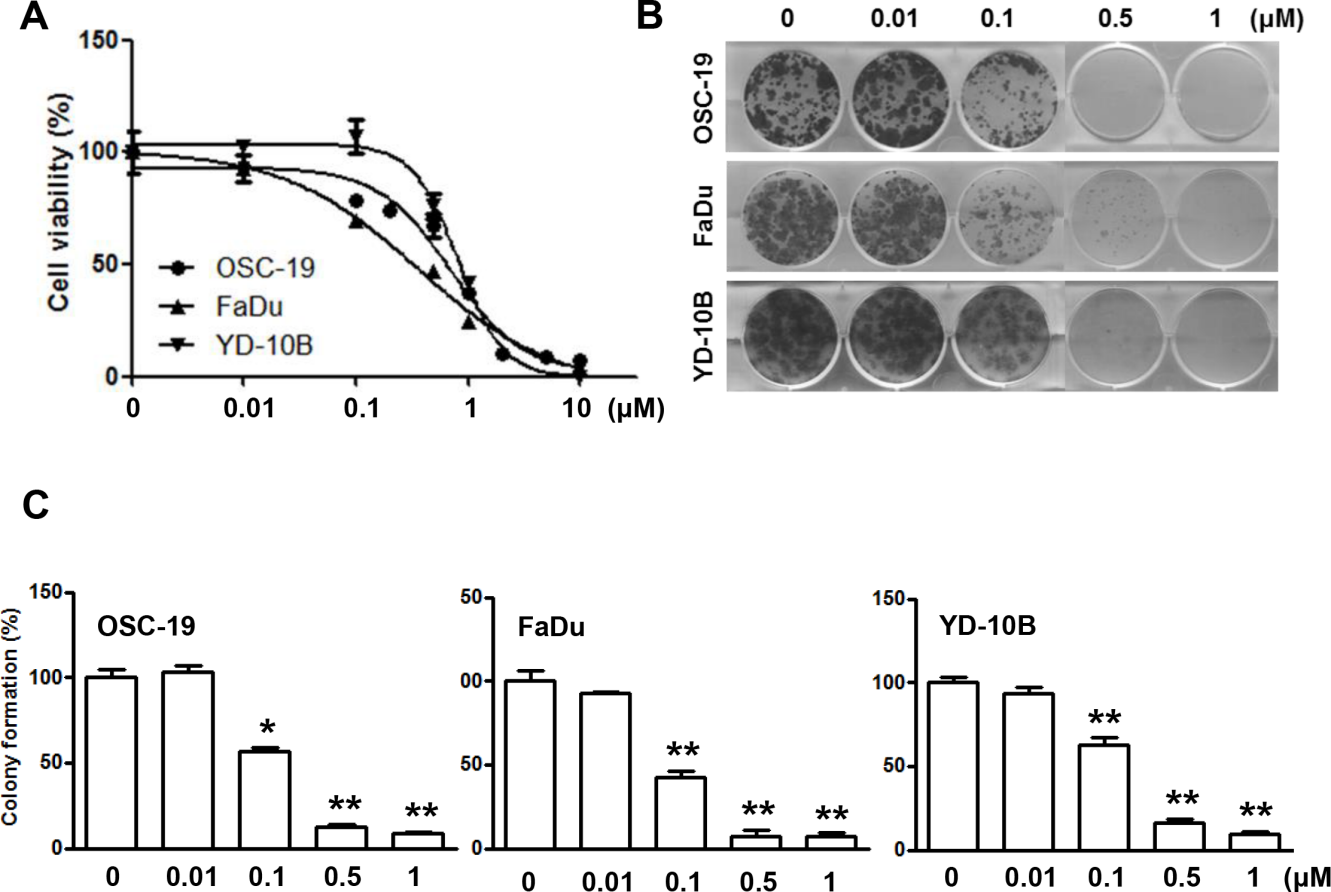
Effects of CDK4/6 inhibitor LY2835219 on cell growth in HNSCC (**A**) Cell viability of OSC-19, FaDu, and YD-10B cells treated with LY2835219 at indicated concentrations. Data are representative of three independent experiments: mean ± SEM. (**B**) Long-term responses to LY2835219 were evaluated in OSC-19, FaDu, and YD-10B cells by colony formation assay. After 10 days of treatment, all cells were fixed and stained using crystal violet. Representative plates are shown. (**C**) Quantification of colony formation. Data represent mean ± SEM (*n* = 3). *P < 0.05; **P < 0.01.

LY2835219 is a novel, selective CDK4/6 inhibitor in clinical development. To demonstrate its impact on HNSCC *in vitro*, we examined the effects of LY2835219 using three HNSCC cell lines (OSC-19, FaDu, and YD-10B). Cells were treated with LY2835219 at concentrations ranging between 0.01 μM and 10 μM for 72 h. This treatment reduced cell viability at HNSCC cells (Figure 1A). The IC_50_ values for LY2835219 ranged from 0.5 μM to 0.7 μM at HNSCC cells. In addition, LY2835219 inhibited colony formation with long term treatment (Figure 1B), indicating that LY2835219 was able to effectively suppress colony formation of HNSCC cells in a dose-dependent manner (Figure 1B).

**Figure 2:**
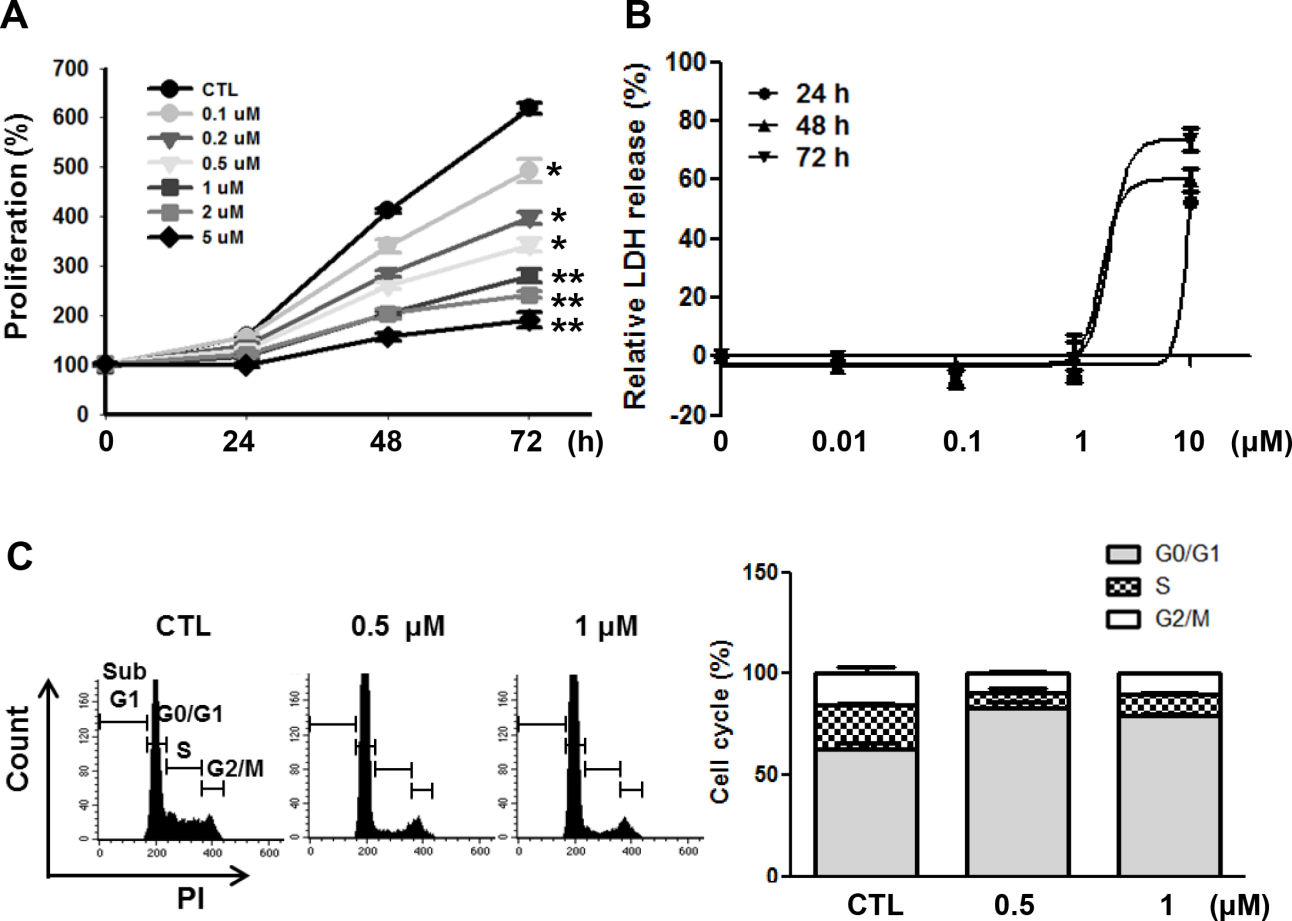
Effects of LY2835219 on cell proliferation and cell cycle in HNSCC (**A**) Growth curves of OSC-19 treated with LY2835219 at indicated concentrations during 72 h. (**B**) LDH release assay. The cytotoxic effect of LY2835219 was determined by detecting LDH release from damaged cells. (**C**) Cell cycle analysis. After 24 h treatment, cell cycle analysis was performed using propidium iodide (PI) staining followed by flow cytometry. Histogram represents the distribution of cells in sub-G1, G0/G1, s and G2/M phases and bar graph represents the percent of G0/G1, S, and G2/M phases of the cell cycle. Data represent mean ± SEM (*n* = 3). *P < 0.05; **P < 0.01.

**Figure 3:**
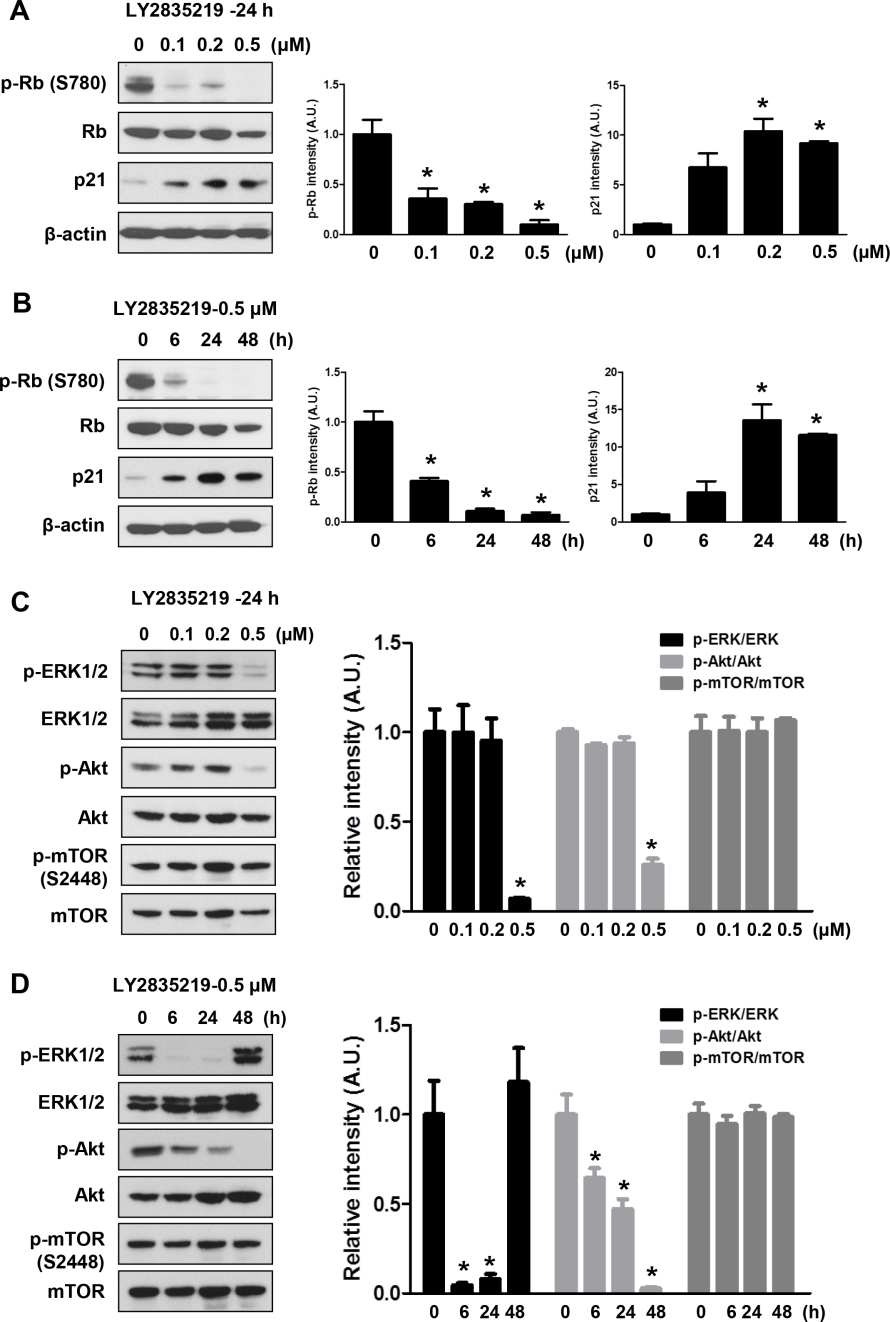
Effects of LY2835219 on RB pathway and intracellular signaling (**A** and **B**) Effects of LY2835219 on RB phosphorylation and p21 expression were evaluated by immunoblotting at indicated concentrations (A) and indicated time points (B). (**C** and **D**). Effects of LY2835219 on phosphorylation of AKT, ERK, and mTOR were evaluated by immunoblotting at indicated concentrations (**C**) and indicated time points (**D**) The graph represents densitometric quantification of immunoblot bands. Data represent mean ± SEM (*n* = 3). *P < 0.05.

To examine the mechanisms of how LY2835219 reduced cell viability, we investigated the effects of LY2835219 on cell proliferation and cell death. As shown in Figure 2A, LY2835219 inhibited cell proliferation in a dose-dependent manner in OSC-19 cells. However, no significant increase of LDH release was observed at a lower concentration (< 1 μM) (Figure 2B). The LDH measurement estimates membrane damage and, therefore, is indicative for cell death. To demonstrate inhibition of CDK4 by LY2835219, we also analyzed the cell cycle. Cell cycle analysis demonstrated cell cycle arrest at G_0_–G_1_ without apoptosis and decreased proportion of S and G_2_–M phase following 24 h of exposure to LY2835219 (Figure 2C). These effects were sustained for 48 h after treatment (data not shown). In line with the ability of LY2835219 to induce G_0_–G_1_ arrest, LY2835219 also reduced RB phosphorylation at Ser780 and increased p21 expression in both a concentration- and time-dependent manner (Figure 3A and 3B). These data suggest the ability of LY2835219 to induce cell cycle arrest and inhibit cell proliferation in HNSCC.

### LY2835219 inhibits Akt and ERK signaling but not mTOR activation

Despite the growth inhibitory effects, LY2835219 was not as effective as single agent as was initially hoped. Thus, we further investigated the molecular mechanism of LY2835219 in HNSCC to find ways to improve the antitumor effects. As the PI3K/AKT/mTOR and MAPK/ERK pathways are known to be targetable oncogenic drivers in HNSCC [4], we examined the effects of LY2835219 on these pathways. OSC-19 cells were treated with 0.1, 0.2, and 0.5 μM LY2835219, and levels of p-AKT (Ser473), p-ERK1/2 (thr202/Tyr204), and p-mTOR (Ser2448) were measured with Western blot analysis. Treatment of cells with LY2835219 inhibited phosphorylation of ERK1/2 and AKT in a dose-dependent manner (Figure 3C). Inhibition of AKT by LY2835219 persisted for 48 h after treatment. In contrast, phosphorylation of ERK had recovered at 48 h (Figure 3D). Unexpectedly, in spite of inhibition of AKT, LY2835219 had no effect on phosphorylation of mTOR at Ser2448 (Figure 3C and 3D), suggesting continuous activation of mTORC1.

### Combination of LY2835219 and mTOR inhibitor shows synergistic effect in HNSCC

**Figure 4:**
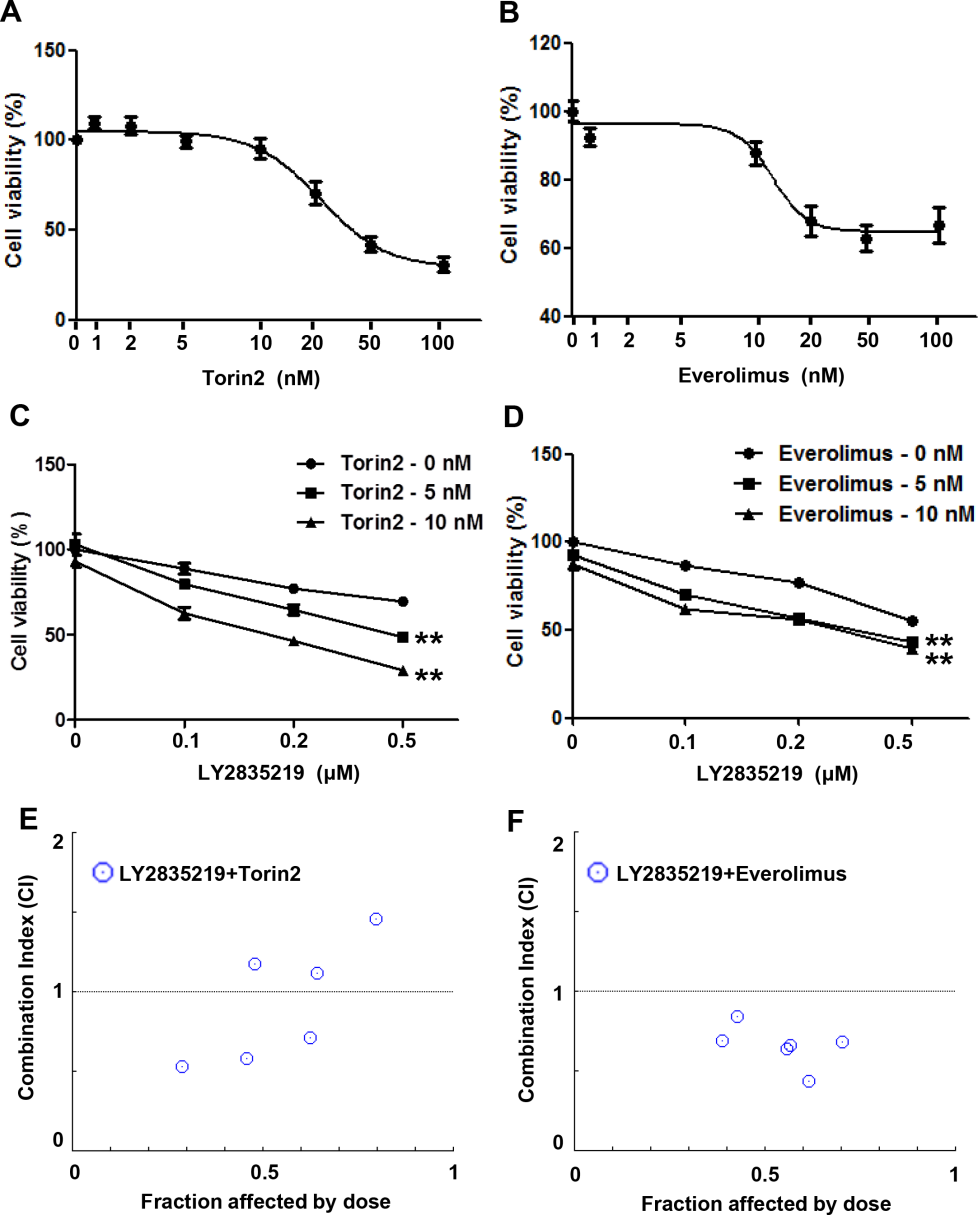
Combined effect of LY2835219 and mTOR inhibitors *in vitro* (**A** and **B**) Sensitivity of OSC-19 cells to torin2 (A) and everolimus (B). (**C** and **D**) Cell viability of OSC-19 cells treated with LY2835219 and torin2 (C) and LY2835219 and everolimus (D). Data represent the mean of six replicates. E and F. Combination index plot of LY2835219 and torin2 (**E**) and LY2835219 and everolimus (**F**) Combination index values that are less than 1 indicated synergistic interactions Data are representative of three independent experiments: mean ± SEM. **P < 0.01.

To test whether sustained mTOR activation causes a moderate effect of LY2835219 in HNSCC, we performed a combinatorial drug treatment using two different mTOR inhibitors, torin2 and everolimus. Everolimus is used in chemotherapeutic regimens against various types of cancers and torin2 exhibits greater anti-proliferative activity in several cancer cell lines [14–16]. Cell viability was assessed after treatment with increasing doses of mTOR inhibitors in the absence and presence of LY2835219. First, we tested the effect of mTOR inhibitors on cell viability in OSC-19 cells (Figure 4A and 4B). As the concentration of up to 10 nM torin2 and everolimus did not significantly affect cell viability, these concentrations were chosen for combination. Intriguingly, bothLY2835219-Torin2 (Figure 4C) and LY2835219-everolimus (Figure 4D) combination significantly reduced cell viability. Based on the CI index, torin2 (Figure 4E) and everolimus (Figure 4F) in combination with LY2835219 showed a synergistic effect (CI < 1) on suppression of cell viability. These effects were dose-dependent in both drugs but everolimus showed a more prominent effect than torin2 at a lower concentration. Thus, we used everolimus for further *in vivo* study.

### LY2835219 inhibits HNSCC tumor growth *in vivo*

**Figure 5:**
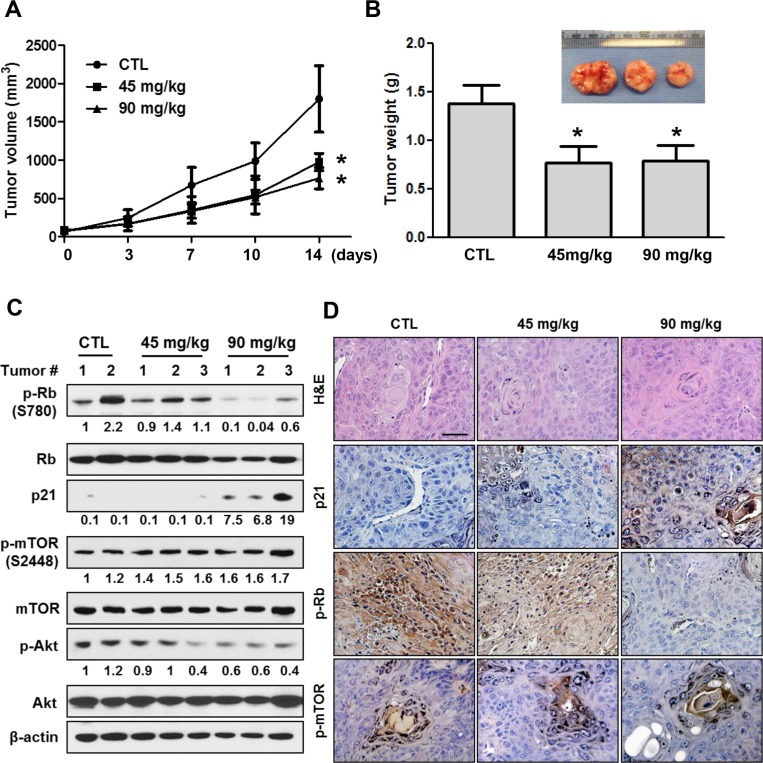
Antitumor activity of LY2835219 in HNSCC xenograft tumor model (**A**) Mice bearing subcutaneous OSC-19 tumors were dosed with vehicle (*n* = 4), 45 mg/kg (*n* = 5), or 90 mg/kg (*n* = 5) LY2835219 once daily for 14 days. Tumor size was measured by caliper twice weekly. (**B**) Tumor weight. Data represent mean volume ± SEM. *P < 0.05; **P < 0.01; compared with respective control group treated with vehicle. (**C**) OSC-19 tumors were collected at the end of experiments. Tumor lysates were prepared and analyzed by immunoblotting using the indicated antibodies. Relative band intensity values are indicated. (**D**) Representative examples of immunohistochemical staining for hematoxylin and eosin (H & E), p21, p-Rb, and p-mTOR in OSC-19 tumor xenografts. Original magnification, x200. Scale bar, 50 μm.

To evaluate the efficacy of LY2835219 in HNSCC *in vivo*, we generated tumor xenografts derived from OSC-19 cells. Mice bearing tumor xenografts were treated once daily for 14 days with LY2835219 (45 mg/kg and 90 mg/kg) or with a vehicle control as previously described [12, 13]. This dosing strategy was tolerable, as no body weight loss or other signs of toxicity were observed. As shown in Figure 5A and 5B, LY2835219 significantly decreased tumor growth of OSC-19 xenografts during treatment. At the end point of the experiment, tumors were harvested and monitored by Western blot and immunohistochemistry using antibodies against p-Rb (Ser780), p21, p-AKT (Ser473), and p-mTOR (Ser2448). Treatment of mice with LY2835219 resulted in a profound dose-dependent inhibition of p-Rb (S780) (Figure 5C and 5D). Concomitant with this change, an increase in p21 expression was observed (Figure 5C and 5D), which may reflect a G_0_–G_1_ arrest by LY2835219. In concordance with *in vitro* results, LY2835219 treatment reduced phosphorylation of AKT, but had no effects on mTOR activation.

### LY2835219 in combination with everolimus causes a cooperative antitumor effect in HNSCC xenograft tumor

**Figure 6:**
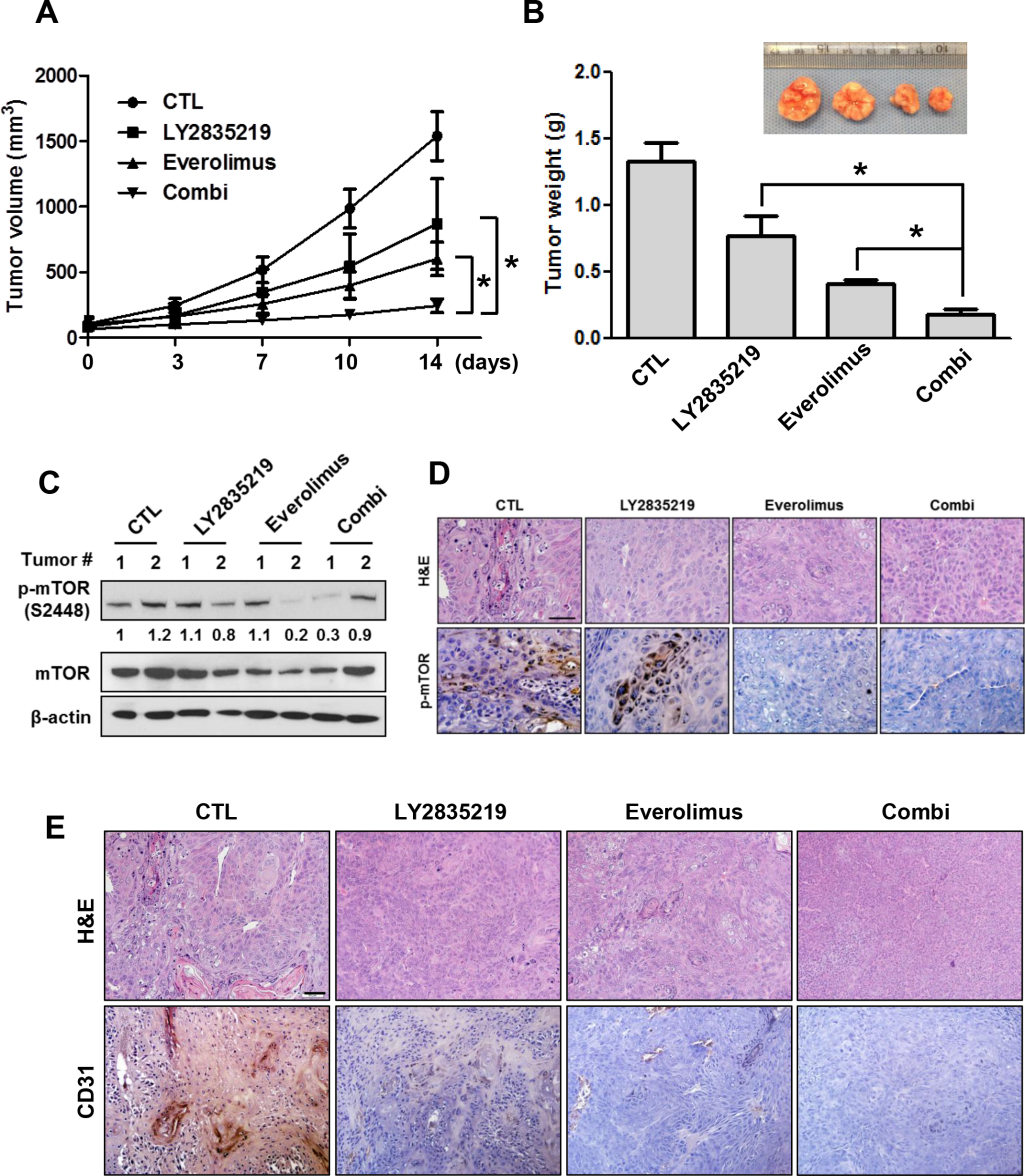
LY2835219 and mTOR inhibitor combination in HNSCC xenograft tumor model (**A**) Mice bearing subcutaneous OSC-19 tumors were dosed with vehicle (*n* =4), 45 mg/kg (*n* = 5), 5 mg/kg everolimus (*n* = 5), or LY2835219 plus everolimus combination (*n* = 5) once daily for 14 days. Tumor size was measured by caliper twice weekly. (**B**) Tumor weight. Data represent mean volume ± SEM. *P < 0.05; **P < 0.01; compared with either LY2835219 or everolimus alone group. (**C**) OSC-19 tumors were collected at the end of the experiment. Tumor lysates were prepared and analyzed by immunoblotting using p-mTOR, mTOR, and β-actin. Relative band intensity values are indicated. (**D**) Representative images of immunohistochemical staining for H & E and p-mTOR. (**E**) Immunohistochemical staining of CD31, an endothelial cell-specific surface marker, in OSC-19 tumor xenografts. Original magnification, x200. Scale bar, 50 μm.

The observed antitumor effect of the LY2835219 and everolimus combination *in vitro* suggested the possibility of similar efficacy *in vivo*. To determine this possibility, the efficacy of LY2835219 and everolimus combination was examined *in vivo*. Mice bearing OSC-19 were treated with vehicle, LY2835219 (45 mg/kg), everolimus (5 mg/kg), or combination of LY2835219 and everolimus. Both LY2835219 and everolimus alone inhibited tumor growth. The combination yielded greater inhibition of tumor growth than either treatment alone (Figure 6A and 6B). No significant changes in body weight were found between four groups during the treatment periods. To validate the action mechanism of LY2835219 and everolimus combination, tumors were further analyzed by Western blot and immunohistochemistry. As expected, phosphorylation of mTOR was effectively inhibited by everolimus combination compared to LY2835219 alone (Figure 6C and 6D). Given CDK4/6 inhibitor has been reported to have an anti-angiogenic activity [17, 18], we performed immunohistochemistry analysis to detect the protein levels of CD31 in the tumor xenograft. Treatment with both LY2835219 and everolimus reduced intratumoral microvessels (CD31) (Figure 6E). Taken together, these results suggest that the combination of LY2835219 and mTOR inhibitor has a synergistic therapeutic effect on HNSCC.

## DISCUSSION

HNSCC displays a handful of widespread genomic alterations that can be evaluated as potential therapeutic targets for personalized medicine. Over-activation of CDK4/CDK6 after *CDKN2A* loss or *CCND1* amplification causes higher RB phosphorylation and drives cell cycle progression [19, 20]. Thus, CDK inhibitor could be an attractive anticancer therapy [10, 21]. LY2835219, an ATP-competitive dual inhibitor of CDK4/6, is an orally bioavailable drug in glioblastoma, acute myeloid leukemia, mantle cell lymphoma, colon and lung xenografts [13, 21]. LY2835219 has also been shown to cross the blood-brain barrier [19, 21]. In preclinical studies, LY2835219 enhances the effects of chemotherapeutic agents, suggesting that it can be used in multidrug therapeutic regimens. Currently, LY2835219 is being investigated in phase I-III clinical trials for advanced non-small cell lung cancer, mantle cell lymphoma, and metastatic breast cancer [20]. In this study, we demonstrated the therapeutic potential of the selective CDK4/6 inhibitor LY2835219 in treating HNSCC.

The data presented here show that CDK4/6 inhibition is a feasible therapeutic strategy in HNSCC. By using LY2835219, we observed RB hypophosphorylation, cell cycle arrest, and dose-dependent tumor growth reduction in HNSCC *in vitro* and *in vivo*. In addition, LY2835219 treatment inhibited angiogenesis in tumor xenograft which is consistent with previous studiesn [17, 18]. We further examined the molecular mechanism of LY2835219 in these cells. As expected, LY2835219 inhibited activation of AKT and ERK, signaling molecules important for the survival and growth of cancer cells. However, treatment of HNSCC cells with LY2835219 had no effects on Ser2448 phosphorylation of mTOR regardless of AKT and ERK inactivation. Such sustained mTOR activity after CDK4/6 inhibitor treatment has been reported in previous study [10]. mTOR is a key downstream signaling component of the PI3K/AKT signaling pathway and ERK was shown to activate mTOR though inhibition of TSC2 [22]. mTOR is a serine-threonine kinase that controls cell survival and growth by regulating various cellular processes. It triggers oncogenic mechanisms including cancer cell survival, cell cycle progression, proliferation, transcription and translation, angiogenesis, invasion, and metastasis [23–25]. Although the persistent activation of AKT/mTOR pathway is a feature of HNSCC [7, 26], activating mutations of mTOR have not been reported previously. Thus, we hypothesized that constant mTOR activation after LY2835219 treatment may cause the modest antiproliferative effect of LY2835219 as a single agent. To prove this, we tested whether dual inhibition of CDK4/6 and mTOR could synergistically induce the antitumor effects in HNSCC.

In the current study, the cogency of cotargeting CDK4/6 and mTOR simultaneously in HNSCC cell lines was confirmed by significant increases in growth inhibition which was observed following treatment with the everolimus in combination with LY2835219 compared with either agent alone. Further, we confirmed everolimus treatment had no effects on Rb phosphorylation (data not shown), and demonstrate that the cotargeting CDK4/6 and mTOR is an attractive targeted therapy for HNSCC. Previous work has indicated that mTOR-specific inhibitors are available and show potent activity against various cancers in preclinical and clinical models [27, 28]. In addition, several studies have suggested that mTOR inhibitor can enhance the efficacy of different chemotherapeutic agents in various cancers [6, 29, 30]. Everolimus, an inhibitor of mTOR in its complex mTORC1, has shown a remarkable antitumor effect against renal cell carcinoma, breast cancer, and neuroendocrine tumors, leading to the food and drug authority (FDA) appoval for the treatment of advanced renal cell carcinoma and metastatic breast cancer. As a results, everolimus is currently part of therapy regimes for metastatic renal cell carcinoma, esophageal cancer, gastric cancer, breast cancer, hepatocellular carcinoma, as well as HNSCC [15, 16, 23, 27, 31]. Torin2 is a second-generation ATP-competitive mTOR inhibitor with a superior pharmacokinetic profile to previous inhibitors. It has been shown to have an antitumor effect in several cancers [14, 32, 33]. The combination of rapamycin, an mTOR inhibitor, with paclitaxel has been shown to significantly increase growth inhibition over paclitaxel alone in HNSCC cell lines [34]. Recently, it was also reported that everolimus can enhance the effect of docetaxel in HNSCC [35]. Furthermore, a phase I studies of mTOR inhibitors plus weekly cisplatin and intensity-modulated radiation therapy for patients with head and neck cancer and a phase I study of mTOR inhibitors plus docetaxel plus cisplatin as induction chemotherapy for patients with locally and/or regionally advanced head and neck cancer are recently completed [36, 37]. These findings suggest that mTOR inhibitors can be combined with current treatment strategies for head and neck cancer, such as radiation or chemotherapy.

In summary, we show the potential use of LY2835219 and combined with mTOR inhibition in HNSCC. Both LY2835219 and mTOR inhibitor suppressed tumor growth of HNSCC as single agent, and the combination of these two agents resulted in synergistic tumor growth inhibition. Thus, the development of an effective therapeutic strategy for HNSCC using a combination of LY2835219 and mTOR inhibitor should be further investigated.

## MATERIALS AND METHODS

### Cell lines and cell cultures

The HNSCC cell lines FaDu and YD-10B were obtained from the Korean Cell Line Bank. Human oral squamous cell carcinoma cell line OSC-19 was obtained from the Japanese Collection of Research Bioresources Cell Bank. FaDu and YD-10B cells were cultured in RPMI1640 and OSC-19 cells were cultured in DMEM. Media were supplemented with 10% FBS and 1% penicillin/streptomycin. Cells were maintained at 37°C in a humidified atmosphere containing 5% CO_2_. All treatments, except LDH assay, were conducted in complete culture medium.

### Chemical reagents and antibodies

LY2835219 was provided by Eli Lilly and Company. LY2835219 was dissolved in dimethyl sulfoxide (DMSO) to a 10 mM concentration and stored in small aliquots at −20°C until further use. Torin-2 was obtained from Tocris Bioscience. Everolimus (RAD001) was obtained from Novartis. DMSO was used as solvent control. Antibodies against p-RB (Ser780), RB, p-Akt (Ser473), Akt, p-ERK1/2 (Thr202/Thy204), ERK1/2, p-mTOR (Ser2448), mTOR, and p21 were purchased from Cell Signaling Technology. Antibodies against β-actin and CD31 were purchased from Santa Cruz Biotechnology.

### Cell viability assay and combination index analysis

Cells were seeded in a 96-well plate, allowed to adhere overnight, and treated with DMSO control (0.1% v/v) or the indicated compounds for 72 h. Cell viability and proliferation were determined using a Cell Counting Kit (Dojindo Molecular Technologies) according to the manufacturer's instructions. The interaction between LY2835219 and mTOR inhibitor was determined using CompuSyn (Combosyn). Combination index (CI) values of 1 indicated and additive drug interaction, whereas a CI of < 1 was synergistic and a CI of > 1 was antagonistic.

### Colony formation assay

Long-term viability assay was performed by colony formation assay. In brief, cells were seeded in 6-well plates and allowed to adhere overnight then treated with DMSO control (0.1% v/v) or LY2835219. Media were changed every 3 days with fresh reagents added. Following 10-day treatment, cells were fixed and stained with crystal violet. Colonies with > 50 cells were quantified using ImageJ (NIH, Bethesda, MA).

### Cell cycle

Cells were treated with DMSO control (0.1% v/v) or LY2835219 for 24 h and harvested. After washing with ice-cold PBS, cells were fixed in 70% ethanol at 4°C. Fixed cells were stained with 10 μg/ml RNase A and 20 μg/ml propidium iodide. DNA content was analyzed by flow cytometry (BD Biosciences).

### Cell death assay

A cell death assay based on the release of lactate dehydrogenase (LDH) was conducted using LDH Cytotoxicity Detection Kit (TaKaRa). Cells were seeded in 96-well plates and allowed to adhere overnight. Cells were treated as indicated concentrations of LY2835219 in low serum (1%) medium according to manufacturer's protocol. After 24 to 72 h treatment, culture medium form each well was collected and transferred into 96-well flat bottom plates. LDH activity was determined by adding equal volumes of reaction mixture to each well and incubating for 30 min. The absorbance was measured at 490 nm using a plate reader and cell death was calculated according to manufacturer's protocol.

### Western blot analysis

Cells and tumor samples were lysed on ice in NP-40 lysis buffer supplemented with a protease and phosphatase inhibitor cocktail (Sigma). Equal amounts of protein were then subjected to SDS-PAGE (NuPAGE 4–12% Bis-Tris Gel; Invitrogen) and transferred to polyvinylidene difluoride (PVDF) membranes. Membranes were then incubated overnight at 4°C with the indicated antibodies and developed by ECL. All blots were quantified with ImageJ.

### Xenograft studies

Six-week-old BALB/c female nude mice were injected subcutaneously with OSC-19 (1 × 10^6^) cells. When tumor sizes reached approximately 100 mm^3^, mice were randomized by tumor size and subjected to each treatment. At least 5 mice per treatment group were included. Each group of mice was dosed via daily oral gavage with vehicle, LY2835219 (45 mg/kg/d or 90 mg/kg/d), Everolimus (5 mg/kg/d), or a combination of both. The LY2835219 was dissolved in 1% HEC in 20 mM phosphate buffer (pH2.0). Tumor size and body weight were measured twice weekly. Tumor volumes were calculated using the following formula: V = (L × W^2^)/2 (L, Length; W, width). Mice were gavaged a final time on day 14 and sacrificed the following day. The tumors were removed for Western blot and immunohistochemistry. All procedures involving animals were reviewed and approved by the Institutional Animal Care and Use Committee (IACUC) at the Samsung Biomedical Research Institute (SBRI). SBRI is an Association of Assessment and Accreditation of Laboratory Animal Care International (AAALAC International) accredited facility and abides by the Institute of Laboratory Animal Resources (ILAR) guidelines.

### Immunohistochemistry

Immunohistochemistry was performed on 5 μm sections of formalin-fixed paraffin-embedded samples. Following deparaffinization and rehydration of the slides, antigen retrieval was performed using Citrate buffer (pH 6.0). After endogenous peroxidase activity was blocked with 3% hydrogen peroxide, sections were incubated at room temperature with indicated antibodies for 2 h. Sections were further processed with horseradish peroxidase-conjugated secondary antibody and then developed with 3,3-diaminobenzidine. Finally, the slides were counterstained with hematoxylin. Images were obtained with an Olympus BX51 microscope.

### Statistical analysis

Data are presented as the mean ± SEM. Statistical analyses were conducted using GraphPad Prism (GraphPad software, La Jolla, CA). *P*-values < 0.05 were considered statistically significant. All reported *P*-values were two-sided.
